# MECP2-related disorders while gene-based therapies are on the horizon

**DOI:** 10.3389/fgene.2024.1332469

**Published:** 2024-02-12

**Authors:** Katherine Allison, Mirjana Maletic-Savatic, Davut Pehlivan

**Affiliations:** ^1^ Royal College of Surgeons in Ireland, School of Medicine, Dublin, Ireland; ^2^ Section of Pediatric Neurology and Developmental Neuroscience, Department of Pediatrics, Baylor College of Medicine, Houston, TX, United States; ^3^ Jan and Dan Duncan Neurological Research Institute at Texas Children’s Hospital, Houston, TX, United States; ^4^ Blue Bird Circle Rett Center, Texas Children’s Hospital, Houston, TX, United States

**Keywords:** MeCP2, Rett syndrome, MECP2 duplication syndrome, dosage sensitive disorders, Allelic disorders, gene based therapies

## Abstract

The emergence of new genetic tools has led to the discovery of the genetic bases of many intellectual and developmental disabilities. This creates exciting opportunities for research and treatment development, and a few genetic disorders (e.g., spinal muscular atrophy) have recently been treated with gene-based therapies. *MECP2* is found on the X chromosome and regulates the transcription of thousands of genes. Loss of *MECP2* gene product leads to Rett Syndrome, a disease found primarily in females, and is characterized by developmental regression, motor dysfunction, midline hand stereotypies, autonomic nervous system dysfunction, epilepsy, scoliosis, and autistic-like behavior. Duplication of *MECP2* causes MECP2 Duplication Syndrome (MDS). MDS is found mostly in males and presents with developmental delay, hypotonia, autistic features, refractory epilepsy, and recurrent respiratory infections. While these two disorders share several characteristics, their differences (e.g., affected sex, age of onset, genotype/phenotype correlations) are important to distinguish in the light of gene-based therapy because they require opposite solutions. This review explores the clinical features of both disorders and highlights these important clinical differences.

## Introduction

The discovery of the genetic bases of many intellectual and developmental disabilities opens new avenues for research and treatment. (Najmabadi et al., 2011; Bamshad et al., 2012; Alazami et al., 2015; Deciphering Developmental Disorders, 2017; Mitani et al., 2021). Some of these genes, including *MECP2, SHANK3,* and *RAI1,* are dosage-sensitive, meaning an increase or decrease in the gene product causes disease. Efficiently recognizing and, importantly, differentiating the phenotypes of these complex diseases is critical for the proper dosing of therapies. In this review, we will focus specifically on *MECP2-*related disorders.

*MECP2* is located at the Xq28 locus of the X chromosome. It binds to methylated DNA and regulates the transcription of thousands of genes. (Chahrour et al., 2008; D'Esposito et al., 1996; Lewis et al., 1992; Meehan et al., 1992; Sirianni et al., 1998). *MECP2* is implicated in X-linked developmental disorders, including Rett Syndrome and MECP2 Duplication Syndrome (MDS). (Amir et al., 1999; Van Esch et al., 2005; Yamamoto et al., 2014). Rett syndrome is most often caused by loss of function and deletion mutations in *MECP2.* It occurs predominantly in females and is characterized by developmental regression between 6 and 18 months, motor dysfunction, midline hand stereotypies, autonomic nervous system dysfunction, epilepsy, scoliosis, and autistic-like behavior. (Hagberg et al., 1983; Hagberg, 2002). *MECP2* loss of function mutations were originally thought to be lethal in males; however, rare hypomorphic or mosaic *MECP2* mutations have been reported in male individuals. (Clayton-Smith et al., 2000; Meloni et al., 2000; Schwartzman et al., 2001; Topcu et al., 2002; Zeev et al., 2002; Kyle et al., 2018). Some deleterious variants in *MECP2* have also been found in patients with X-linked intellectual disability (Lindsay et al., 1996; Dotti et al., 2002; Gomot et al., 2003) and autism. (Carney et al., 2003). MDS is caused by a duplication (or triplication) of Xq28 that includes *MECP2*. It is primarily seen in males but can be seen in females with abnormal X chromosome inactivation (XCI) patterns or translocations. (Van Esch, 2008; Auber et al., 2010; Makrythanasis et al., 2010; Shimada et al., 2013a; Shimada et al., 2013b; Fieremans et al., 2014; Scott Schwoerer et al., 2014). MDS presents with developmental delay, early hypotonia, gastrointestinal problems, refractory epilepsy, neurobehavioral trait and recurrent respiratory infections. (Van Esch, 2008). Broad spectrum of phenotypes considered to be caused by MECP2 are summarized in Table 1.

**TABLE 1:** MECP2 related phenotypes published in the literature.

MECP2 related phenotypes	OMIM number	Citations
Rett Syndrome Typical/Atypical/Preserved Speech Variant	312,750	PMID: 10508514, PMID: 19034540, PMID: 16630165, PMID: 15857422
MECP2 Duplication Syndrome	300,260	PMID: 15689435, PMID: 16080119, PMID: 17172942, PMID: 21964572
Severe neonatal encephalopathy	300,673	PMID: 11930274, PMID: 11402105
X-linked syndromic intellectual developmental disorder-13	300,055	PMID: 12615169, PMID: 16966553
Autism	300,496	PMID: 11106359, PMID: 15615769, PMID: 23352163
Systemic Lupus Erythematosus	NA	PMID: 18320046
Schizophrenia	NA	PMID: 24776741
Multiple Sclerosis	NA	PMID: 28604632, PMID: 36232761

NA: not assigned.

**FIGURE 1:**
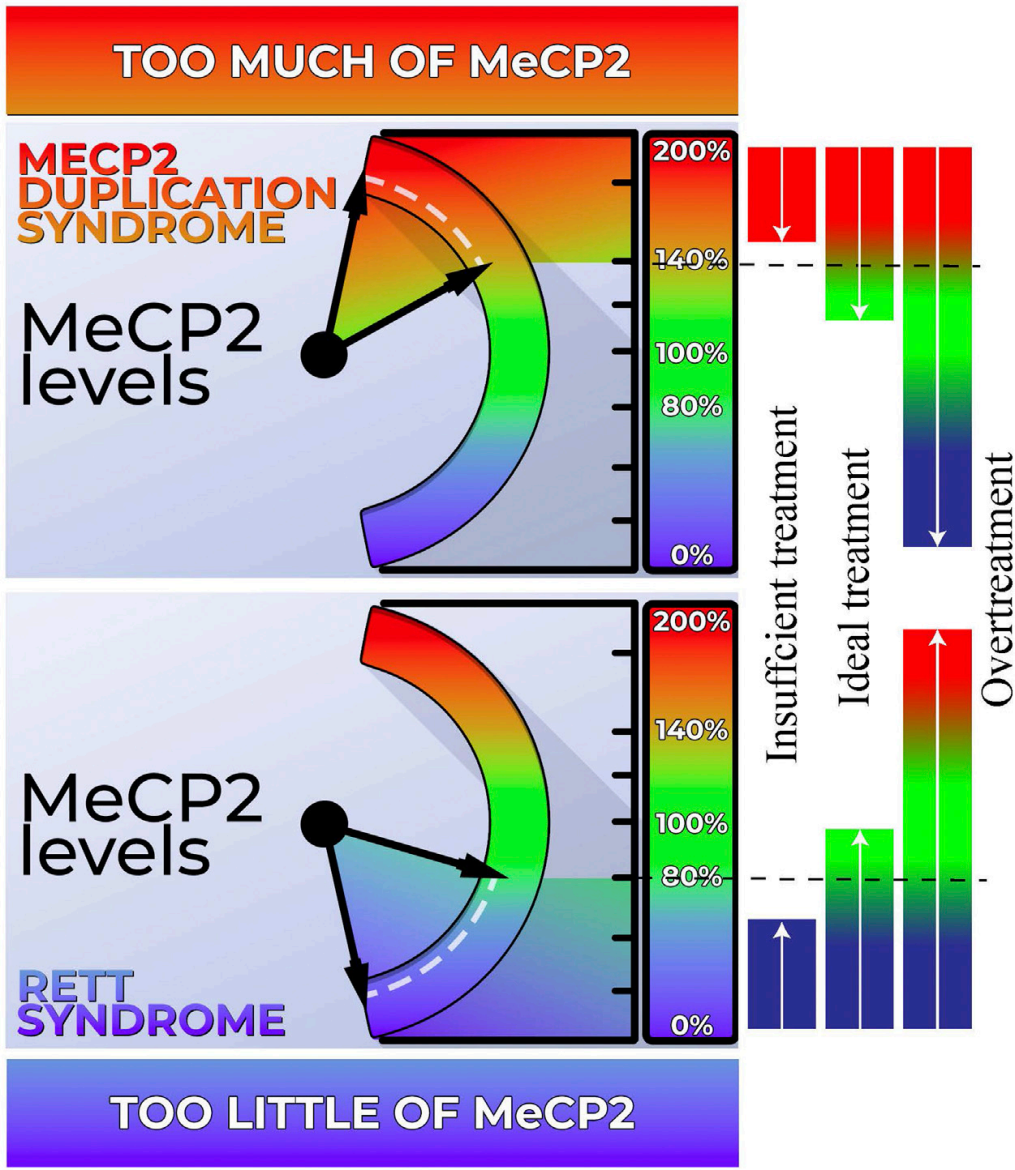
Dosage sensitivity of *MECP2*. Left Panel: Our body exhibits a high sensitivity to MECP2 levels. When MECP2 levels fall below approximately 80%, individuals display features characteristic of Rett syndrome (RTT). Conversely, MECP2 levels exceeding 140% result in the manifestation of MECP2 Duplication Syndrome (MDS). Right Panel: Both RTT and MDS hold promise for genetic-based treatments. However, achieving the ideal dosage is crucial for effectively addressing these conditions. Administering an excessive amount of genetic-based treatment may inadvertently transform RTT into MDS or *vice versa*. Conversely, inadequate treatment may not sufficiently ameliorate the phenotype.

There is great potential for gene-based therapies in neurogenetic disorders. A few genetic disorders (e.g., spinal muscular atrophy and Duchenne muscular dystrophy) have recently been successfully treated with gene-based therapies. However, dosage sensitivity of *MECP2* (Figure 1) complicates the development of treatments since over- or under-correcting the genetic aberration can lead to the opposite disorder or insufficient treatment, respectively. Thus, there is an ongoing effort to develop biomarker/s that correlate with MECP2 protein levels and can be used to titrate treatments. While Rett Syndrome and MDS share many clinical features, there are subtle clinical differences despite their origin from the same gene and neurodevelopmental disease category. Phenotypic differences are important to establish, especially in the light of upcoming gene-based therapy when the two disorders require opposite solutions. This review explores the clinical features of both disorders and highlights these important clinical differences.

### Clinical presentation of Rett syndrome

Rett syndrome was first described by Dr. Andreas Rett in 1966, (Rett, 1966), and then further detailed by Dr. Bengt Hagberg in 1983. (Hagberg et al., 1983). Rett syndrome is a neurodevelopmental disorder that primarily affects females (Hagberg et al., 1983) and has a prevalence of 1:10,000-15,000. (Hagberg, 1985). The majority of cases are caused by mutations in *MECP2*. (Amir et al., 1999). Rett syndrome is characterized by four main stages. Stage I is early onset disease and resembles relatively normal early development. The signs and symptoms of developmental delay are subtle and easily overlooked during this stage. Stage II is characterized by rapid developmental regression where affected children lose many acquired skills. This stage starts between 6–18 months of age and can last for a few months to few years. The third stage is the pseudostationary or plateau phase. The phenotype stabilizes and can remain unchanged for several years to decades. Finally, in Stage IV, there is late motor deterioration. (Hagberg et al., 1985). The four core diagnostic criteria for classic Rett syndrome are hand stereotypies, partial/complete loss of hand function, partial/complete loss of language, and impaired gait. A diagnosis of atypical Rett syndrome requires two of the core criteria to be present in the setting of regression along with 5 of 11 supportive criteria that includes breathing abnormalities when awake (breath holding or hyperventilation), bruxism while awake, disrupted sleep pattern, growth failure, cold and small extremities, peripheral vasomotor disturbances, scoliosis/kyphosis, abnormal muscle tone, inappropriate laughing/screaming spells, decreased response to pain, and intense gaze (eye pointing). (Neul et al., 2010).

The earliest signs of Rett syndrome are developmental regression and loss of early acquired skills including language, fine motor skills, and purposeful hand movements. (Hagberg et al., 1983; Hagberg, 2002). Head circumference growth may also slow down, leading to microcephaly. These features usually present at 1–2 years of age. (Hagberg et al., 1983; Hagberg, 2002).

Midline hand stereotypies are a defining characteristic of Rett syndrome. They normally appear after developmental regression and include midline clapping/tapping, hand mouthing, and wringing/washing. (Stallworth et al., 2019; Ferreira and Teive, 2020). While hand stereotypies remain prevalent over time, the nature of the stereotypies may change. Certain behaviors, like clapping/tapping and hand mouthing, decrease over time while wringing/washing does not change. (Stallworth et al., 2019). The number of hand stereotypies and the severity of mouthing is higher in the pediatric population. (Ferreira and Teive, 2020). Hand stereotypies also correlate with the type of *MECP2* mutation. Individuals with R106W, R168X, R255X, R270X, insertion, and large deletion mutations develop hand stereotypies earlier, (Stallworth et al., 2019), and they display the highest frequency and largest variety of hand movements. (Ferreira and Teive, 2020). Although clinical severity worsens with decreased hand function, hand stereotypies have little correlation with overall clinical severity as assessed with the Clinical Severity Scale (CSS) and Motor Behavior Assessment (MBA). (Stallworth et al., 2019; Ferreira and Teive, 2020). Other movement disorders, including tremor, ataxia, hypertonia, and dystonia, are also seen in Rett patients. (Brunetti and Lumsden, 2020; Young et al., 2022).

Autonomic nervous system (ANS) dysfunction is thought to underlie many clinical features in Rett Syndrome, including cardiac arrhythmias and respiratory irregularities, cold and discolored extremities, and excessive drooling and sweating. Patients have irregular patterns of hyperventilation and breath-holding while awake. (Lugaresi et al., 1985; Weese-Mayer et al., 2006). The breath holds occur frequently and spontaneously without any meaningful pattern. Breath holding also leads to tachycardia in Rett syndrome patients. (Weese-Mayer et al., 2006). Patients may exhibit prolonged QT intervals on ECGs (Ellaway et al., 1999) and have reduced heart rate variability. (Lindsay et al., 1996; Carroll et al., 2020). ANS dysregulation of respiratory and cardiac functions are important to monitor because 20% of deaths in Rett patients occur suddenly while awake. (Kerr et al., 1997). Patients have also been reported to have very cold extremities that periodically sweat profusely. (Hagberg, 2002).

Gastrointestinal (GI) dysfunction is another common clinical feature of Rett syndrome, with constipation being one of the most prevalent symptoms. The spectrum of GI problems is broad including growth failure, chewing and swallowing difficulty, gastroesophageal reflux disorder (GERD), gas bloating, constipation and biliary tract disease. (Motil et al., 1999; Prior et al., 2010; Motil et al., 2012; Baikie et al., 2014; Motil et al., 2019). *MECP2* is expressed in the enteric nervous system, and the dysmotility seen in many patients may be due to dysfunction of the enteric nervous system. (Wahba et al., 2015). Microbiome changes have also been reported in Rett syndrome, which may contribute to GI dysfunction. Patients have increased GI inflammation, reduced microbiotic richness and bacterial metabolic end-products, and altered short-chain fatty acid production. (Strati et al., 2016; Thapa et al., 2021). Thapa et al. found microbiotic differences between Rett patients based on pubertal status, clinical severity scores, and diet. Patients who were post-puberty, had worse clinical severity scores, or only consumed formula had decreased microbiotic richness and diversity. (Thapa et al., 2021). However, there were no differences between patients based on stool consistency or frequency. (Strati et al., 2016; Thapa et al., 2021). GI dysfunction can cause significant pain in patients, (Barney et al., 2015), making it an important feature to address. GI health questionnaires have been developed for Rett syndrome and can be used in severity assessments and clinical trials. (Motil et al., 2021).

Epilepsy is a frequent component of Rett syndrome, and it is associated with more severe disease. (Tarquinio et al., 2017). Approximately 90% of patients develop seizures at some point during their lifetime, (Tarquinio et al., 2017), with seizures appearing around 3 years of age. (Glaze et al., 2010; Nissenkorn et al., 2010; Tarquinio et al., 2017). Seizure type is variable and appears to be evenly split between focal-onset and generalized-onset seizures. (Tarquinio et al., 2017). Tarquinio, et al. examined the longitudinal course of epilepsy with data from the Rett Syndrome Natural History study and found that seizure patterns commonly change over time and that most patients go through cycles of remission and relapse. (Tarquinio et al., 2017). The patients who did not go through these cycles either developed intractable epilepsy or went into chronic remission. While smaller cohort studies found that ∼36% of patients with epilepsy did not respond to medication, (Vignoli et al., 2012a; Anderson et al., 2014), Tarquinio, et al. found that only 16% of patients had intractable epilepsy. Patients who experience cycles of remission and relapse tend to develop seizures later than patients who develop intractable epilepsy or enter chronic remission. (Tarquinio et al., 2017).

Scoliosis is a common and sometimes severe feature of Rett syndrome. About half of patients develop scoliosis in adolescence, and approximately 20% of those cases require surgical correction. (Killian et al., 2017; Rodocanachi Roidi et al., 2022; Menachem et al., 2023). Scoliosis is correlated with a more severe clinical presentation. Puberty is a risk factor for developing scoliosis while patients who can walk independently have a decreased risk. (Killian et al., 2017).

Rett patients are often described as having autistic behavior. (Hagberg et al., 1983; Hagberg, 2002). The US Natural History Study looked at the behavioral profile of Rett patients and found that essentially all patients displayed internalizing behavior (e.g., crying, anxiety, loneliness) to a moderate degree. External behaviors such as hitting occurred less frequently and were less severe. Over time, externalizing behaviors improved while internalizing behavior became more frequent. (Buchanan et al., 2019).

Impaired sleep pattern is one of the minor criteria for Rett syndrome. Several studies showed that sleep problems are present in 80% of individuals with Rett. (Coleman et al., 1988; Young et al., 2007; Wong et al., 2015). Some studies also reported a high prevalence of night laughing (77%) and night screaming (49%) accompanying the sleep disturbances. (Coleman et al., 1988; Young et al., 2007). Boban et al. found both sleep maintenance (75%) and sleep initiation (60%) are the most prevalent sleep problems for individuals with Rett. (Boban et al., 2016).

Finally, phenotype/genotype correlations have been identified in Rett syndrome. These are important to elucidate for future therapies. Over 240 disease-causing mutations have been reported in *MECP2,* and 54% mutation-positive cases are caused by 1 of 8 mutations (T158M, R168X, R255X, R270X, R294X, R306C, R133C, and R106W). (Christodoulou et al., 2003). The variety of mutations causes significant clinical heterogeneity, as seen in differences in severity and age of onset. (Christodoulou and Weaving, 2003). Missense mutations typically cause milder phenotypes, followed by late truncating mutations. Early truncating mutations cause more severe phenotypes. The degree of X chromosome inactivation in individual patients also affects the phenotype. (Weaving et al., 2003).

### Clinical presentation of MECP2 duplication syndrome

In 1999, Lubs et al. performed a linkage study on a family with five affected males with developmental and intellectual disabilities and mapped the causative gene to the Xq28 region. (Lubs et al., 1999). It took 6 years to identify MECP2 as the causative gene for Lubs type X-linked intellectual developmental disorder (the initial name for MDS). (Van Esch et al., 2005; del Gaudio et al., 2006; Meins et al., 2005). MDS predominantly affects males, and the most prevalent features include severe to profound developmental delay/intellectual disability (DD/ID), hypotonia, dysmorphic facial features, refractory epilepsy, recurrent respiratory infections, GI dysfunction, and autistic behavior. (Van Esch et al., 2005; Ramocki et al., 2009; Ramocki et al., 2010; Yi et al., 2016; Lim et al., 2017; Miguet et al., 2018; Giudice-Nairn et al., 2019; Pascual-Alonso et al., 2020). Only a few hundred cases have been published globally, (Van Esch, 2008), and there is varying information on the incidence and prevalence of MDS. Guidice-Nairn et al. reported the incidence of MDS to be 1 in 100,000 live male births in Australia. (Giudice-Nairn et al., 2019). Microarray analysis on ∼5,300 patients with developmental, behavioral, or congenital abnormalities indicated that 4.4% had pathological changes in subtelomeric regions, and sixteen of these patients had Xq28 duplications. (Shao et al., 2008). Lugtenberg et al. found that ∼1% of patients with unexplained X-linked DD/ID had Xq28 duplications that included *MECP2*. This increased to 2% of males with severe, progressive encephalopathy. (Lugtenberg et al., 2009).

DD/ID and little to absent speech were almost universal clinical features in cohort studies. (Van Esch et al., 2005; Ramocki et al., 2010; Yi et al., 2016; Lim et al., 2017; Miguet et al., 2018; Giudice-Nairn et al., 2019; Pascual-Alonso et al., 2020). Most children with MDS can sit independently, (Van Esch et al., 2005; Lim et al., 2017; Giudice-Nairn et al., 2019), but the proportion of patients who learn to walk independently varies widely among studies. Lim et al. reported that only ∼25% of their patients could walk independently, (Lim et al., 2017), while Giudice-Nairn et al. found that over 50% of patients achieved independent walking. (Giudice-Nairn et al., 2019). Miguet et al. found that 20% of their cohort were unable to walk independently. (Miguet et al., 2018). Patients who did learn to walk learned much later (Yi et al., 2016; Miguet et al., 2018), and they walked with a crouching gait. (Van Esch et al., 2005; Miguet et al., 2018; Giudice-Nairn et al., 2019). Some patients later lost their ability to walk. (Lim et al., 2017; Miguet et al., 2018; Giudice-Nairn et al., 2019). Motor function and functional skills degrade over time. (Peters et al., 2021).

Epilepsy is extremely common in MDS and is a significant feature contributing to the burden on caregivers of individuals with MDS. The incidence of epilepsy in case studies is heavily dependent on the age of the patient and the length of follow up. Many studies report that approximately half of their patients develop seizures. (Yi et al., 2016; Lim et al., 2017; Miguet et al., 2018; Giudice-Nairn et al., 2019; Marafi et al., 2019; Pascual-Alonso et al., 2020). However, Vignoli et al. found that >90% of patients had epilepsy by the time they reached adolescence, (Vignoli et al., 2012b), and we recently showed that epilepsy is a dynamic feature and becomes universal with age. (Ak et al., 2022a). Seizure onset occurs later than in Rett syndrome, usually between the ages of 6–9. MDS patients have abnormal background EEGs, and while there is no universal seizure or EEG pattern, atonic seizures are the most common. (Vignoli et al., 2012b; Marafi et al., 2019; Lorenzo et al., 2021). Most of these patients have drug-resistant epilepsy, (Vignoli et al., 2012b; Lim et al., 2017; Miguet et al., 2018; Marafi et al., 2019; Lorenzo et al., 2021), and developmental regression tends to coincide with the onset of epilepsy. (Miguet et al., 2018; Giudice-Nairn et al., 2019; Marafi et al., 2019; Pascual-Alonso et al., 2020). Epilepsy contributes significantly to the morbidity of the disease, and further research is required to fully elucidate this phenotype.

Recurrent respiratory infections, mainly pneumonia, frequently reported in MDS patients and are the most common cause of early death. (Van Esch et al., 2005; Yi et al., 2016; Lim et al., 2017; Miguet et al., 2018; Giudice-Nairn et al., 2019; Pascual-Alonso et al., 2020; Ak et al., 2022b). Some patients have an IgA and IgG_2_ deficiency and low antibody titers against pneumococci, suggesting an impairment in humoral immunity. (Bauer et al., 2015; Bauer et al., 2018). The inclusion of *IRAK1* in the duplication, which encodes a protein that acts on the Toll-like receptor pathway, may play a role. (Bauer et al., 2015). The most common infections, in descending order, are upper respiratory infections, lung infections, urinary tract infections and sepsis. (Ak et al., 2022b). Recurrent aspiration pneumonia has also been reported. (Kirk et al., 2009; Honda et al., 2012; Nascimento et al., 2014; Deshwar et al., 2018). MDS patients frequently have feeding problems, putting them at higher risk of aspiration. (Ramocki et al., 2010; Yi et al., 2016; Lim et al., 2017; Miguet et al., 2018; Giudice-Nairn et al., 2019; Pascual-Alonso et al., 2020). Given the prominence of severe infections and lack of literature confirming the role of humoral immune deficiency, this area requires serious attention.

Autism has been found to be universally present in individuals with MDS. (Ramocki et al., 2009). Peters et al. compared the core symptoms of autism in individuals with MDS and those with idiopathic autism in a small cohort (N = 10 in each group). They demonstrated that hyposensitivity to pain/temperature is a characteristic of MDS, while core behavioral features (social affect, repetitive behavior) are similar in both MDS and idiopathic autism. (Peters et al., 2013). Hand stereotypies are common and include hand flapping at sides, body rocking and head movements. Rarely, MDS individuals have midline hand movements such as wringing. (Ramocki et al., 2009; Ramocki et al., 2010; Yi et al., 2016; Lim et al., 2017; Miguet et al., 2018; Pascual-Alonso et al., 2020). Other frequently seen behaviors include bruxism, delayed pain response, and passivity. (Ramocki et al., 2009; Lim et al., 2017; Miguet et al., 2018; Peters et al., 2019; Ak et al., 2022b). However, self-mutilation, irritability, overactivity or other behavioral problems are not commonly reported in individuals with MDS. (Peters et al., 2019; Ak et al., 2022b).

Dysmorphic features have been described in several studies. The most commonly reported features included hypotonic face, large ears, flat nasal bridge, posterior skull asymmetry, midface hypoplasia, and deep-set eyes. Some patients also have small, flat feet and long, thin, tapered fingers. (Van Esch et al., 2005; Ramocki et al., 2010; Yi et al., 2016; Miguet et al., 2018). Finally, there have been a few reports of urogenital abnormalities and cryptorchidism. (Yi et al., 2016; Miguet et al., 2018; Giudice-Nairn et al., 2019; Ak et al., 2022b).

Gastrointestinal problems, including feeding difficulty, GERD and constipation have commonly been reported in individuals with MDS. (Lim et al., 2017; Miguet et al., 2018; Peters et al., 2019; Pascual-Alonso et al., 2020). Constipation was among the top six concerns for which caregivers sought treatment. (Ak et al., 2022b). Pehlivan et al. conducted a detailed study of gastrointestinal problems in MDS and compared their findings with those in Rett syndrome. In addition to confirming the high frequency of constipation (72%–89%), feeding difficulties (70%) and GERD (51%), the authors also demonstrated a bimodal distribution of GERD and gastrostomy feeding (higher in 0–3 years and >12 years old age groups). Furthermore, the authors identified biliary tract disease in 5% of individuals with MDS, a finding not previously reported. (Pehlivan et al., 2023).

While insomnia is common in Rett, it has not been extensively studied in MDS. Peters et al. reported sleep problems in 43% of individuals with MDS. (Peters et al., 2019). Ak et al. provided a breakdown of sleep problems with the following frequencies: 48% experienced difficulty with sleep maintenance, 35% had problems with sleep initiation, 25% struggled with waking up in the morning and 48% exhibited snoring. (Ak et al., 2022b).

Scoliosis is the most common musculoskeletal problem reported in MDS. While Miguet et al. found scoliosis in 23 out of 43 (53%) MDS individuals, (Miguet et al., 2018), Peters et al. reported a lower frequency of scoliosis in 9 out of 48 (19%) MDS individuals in the US Natural History Study. (Peters et al., 2019). Lim et al. demonstrated that scoliosis is progressive and becomes more evident with age. (Lim et al., 2017). Ak et al. expanded upon musculoskeletal findings and observed scoliosis in 37%, joint laxity/dislocation in 29%, bone fracture in 10% and contracture in 10% of individuals with MDS. (Ak et al., 2022b).

Miguet et al. reported genitourinary anomalies in 23/53 (43%) of individuals with MDS, distributed as follows: cryptorchidism 26%, micropenis 9%, ureteral dilation 9% and bladder dilation/hypertrophy 5%. (Miguet et al., 2018). Ak et al. reported the following frequencies: cryptorchidism 39%, micropenis 25%, urinary retention 31% and kidney anomaly 4%. (Ak et al., 2022b).

Visual abnormalities are also commonly reported, including hypermetropia 54%, amblyopia 17%, myopia 14%, strabismus 13%, nystagmus 6% and astigmatism 4%. (Miguet et al., 2018; Ak et al., 2022a). Additionally, Ak et al. reported a high frequency of eczema 51% and allergy 40%. The authors additionally reported heart problem 17%, lung problems 17% and hearing loss 11% without providing further details. (Ak et al., 2022a).

Finally, each patient has a different size duplication, though they share a common duplicated region that includes *MECP2* and *IRAK1*. (Van Esch et al., 2005; Carvalho et al., 2009; Yi et al., 2016). There are several nearby genes in the Xq28 locus including *L1CAM, RAB39B, GDI1, FLNA* and *HCFC1*, in which loss of function mutations cause neurodevelopmental disorders. (Van Esch et al., 2005; Yamamoto et al., 2014; Yi et al., 2016; Peters et al., 2019). Importantly, duplications of *RAB39B* (∼1.1 Mb distal to *MECP2*) and *GDI1* (∼300 kb distal to *MECP2*) are independently associated with intellectual disability, thus creating the potential for a patient to be misdiagnosed with MDS. (Vandewalle et al., 2009; Vanmarsenille et al., 2014; Yamamoto et al., 2014). MDS patients with a *RAB39B* duplication have more severe microcephaly and motor impairments. (Peters et al., 2019). The potential role of the other nearby genes is unclear, although *FLNA* has been associated with more severe GI symptoms. (Clayton-Smith et al., 2009). Work done in mice models over-expressing only wild-type *MECP2* suggests that the phenotype is primarily due to *MECP2* duplication since these mice recapitulate many MDS features, including spasticity, epilepsy, and stereotypic movements. (Collins et al., 2004; Luikenhuis et al., 2004; Samaco et al., 2012). Despite the variability in duplication size, a genotype/phenotype correlation has been difficult to establish. Conflicting conclusions have been made on whether the size of the duplication affects the severity of symptoms. Miguet, et al., Lugtenberg, et al., and Yi, et al. did not find any genotype/phenotype correlations. (Lugtenberg et al., 2009; Yi et al., 2016; Miguet et al., 2018). Peters, et al. found that larger duplication size and gene content contributes to phenotype severity especially if the duplication includes *RAB39B*. (Peters et al., 2019). Pascual-Alonso, et al. found that duplications translocated outside of the X chromosome cause more severe phenotypes. (Pascual-Alonso et al., 2020). Triplication cases have also been identified, (Mayo et al., 2011; Honda et al., 2012; Tang et al., 2012; Signorini et al., 2016), and these patients have more severe disease. Respiratory insufficiency, dysphagia, hearing loss, and cardiac defects are more common than in patients with duplications. Additional features like polyhydramnios and intestinal pseudo-obstruction have also been found in triplication patients. (Carvalho et al., 2011).

### Comparison

Rett syndrome and MDS are neurodevelopmental disorders primarily caused by aberrations in *MECP2*. These two disorders share many clinical features, including DD/ID, problems with gross and fine motor skills, absent/delayed speech, scoliosis, epilepsy, behavioral abnormalities, hand stereotypies, insomnia and GI dysfunction. However, despite the similarities, there are important differences in the severity and age of onset of certain clinical features (Table 2).

**TABLE 2:** Clinical comparison between MECP2 Duplication and Rett Syndrome.

Clinical feature	MECP2 duplication syndrome	Rett syndrome
Stereotypies	At sides, not interfering with hand function	Midline, interferes with hand function
Tremor	-	++ (especially male individuals with MECP2 single nucleotide variants)
Epilepsy	>90% refractory, age of onset 6–9 years	<20% refractory, age of onset 3–4 years
Gait abnormality	++ crouching gait	+++ dyspraxic
Vasomotor Disturbance	+	++
Periodic Breathing	−/+	+++
Growth failure	-	+++
Recurrent Infections	+++	-
Gas/bloating	−/+	+++

The most obvious difference is that Rett syndrome primarily affects females while MDS is much more common in males. Males with *MECP2* single nucleotide or indel mutations display a wide range of clinical severity, including severe neonatal encephalopathy, classic Rett syndrome, and more mild neuropsychiatric symptoms. (Clayton-Smith et al., 2000; Meloni et al., 2000; Schwartzman et al., 2001; Topcu et al., 2002; Zeev et al., 2002; Kyle et al., 2018). Classic Rett syndrome can occur in males if they have Klinefelter syndrome (Schwartzman et al., 2001) or are mosaic for the *MECP2* mutation. (Clayton-Smith et al., 2000; Topcu et al., 2002). Most females who are heterozygous for a *MECP2* duplication are asymptomatic due to skewed X chromosome inactivation (XCI). (Van Esch, 2008). However, neuropsychiatric symptoms (e.g., depression, anxiety, autistic features) have been described in some asymptomatic females. (Ramocki et al., 2009). Rarely, females will display classic MDS symptoms, which can also be attributed to skewed XCI. This occurs in patients with translocation to an autosome or other unknown mechanism. (Auber et al., 2010; Makrythanasis et al., 2010; Shimada et al., 2013a; Shimada et al., 2013b; Fieremans et al., 2014; Scott Schwoerer et al., 2014; San Antonio-Arce et al., 2016).

The timing of symptom development differs between the two disorders. Clinically, children with Rett syndrome appear to develop normally for the first 6–18 months of life before experiencing developmental delay and regression. (Hagberg et al., 1983; Hagberg, 2002). Children with MDS show developmental delay early on, and regression occurs later in life. (Van Esch et al., 2005; Yi et al., 2016; Lim et al., 2017; Miguet et al., 2018; Giudice-Nairn et al., 2019; Pascual-Alonso et al., 2020). Regression is most often seen in MDS individuals who have recurrent respiratory infections, develop epilepsy, or have refractory epilepsy. (Marafi et al., 2019).

While epilepsy is common in both disorders, its presentation is very different. It is more prevalent in Rett syndrome, with ∼90% of patients developing seizures at some point in their life. However, it is usually manageable with medication, and most patients go through cycles of remission and relapse. (Tarquinio et al., 2017). Prevalence of epilepsy ranges between 43%–59% of MDS patients. (Ramocki et al., 2010; Van Esch, 2012; Lim et al., 2017; Miguet et al., 2018; Marafi et al., 2019; Peters et al., 2021). However, recent studies have shown that epilepsy becomes almost universal as MDS individuals get older. (Vignoli et al., 2012b; Ak et al., 2022a). Many of these MDS patients do not respond or poorly respond to anti-seizure medications and ultimately develop epileptic encephalopathy. (Marafi et al., 2019). Finally, the age of onset differs between the two syndromes. Rett patients typically develop seizures between the ages of 3–4, (Glaze et al., 2010; Nissenkorn et al., 2010; Tarquinio et al., 2017), while epilepsy appears between the ages of six to nine in MDS patients. (Lim et al., 2017; Miguet et al., 2018; Giudice-Nairn et al., 2019; Marafi et al., 2019).

ANS abnormalities occur in both disorders, though it is more prominent in Rett syndrome. These patients display cardiac and respiratory anomalies, cold hands and feet, and excessive sweating. (Lugaresi et al., 1985; Ellaway et al., 1999; Hagberg, 2002; Weese-Mayer et al., 2006). MDS patients may also have breathing abnormalities (Peters et al., 2019) and cold extremities, but in our experience, these features are much milder than what is observed in Rett syndrome.

MDS patients develop severe recurrent respiratory infections, possibly due to the involvement of *IRAK1* (Bauer et al., 2015) or an increased risk of aspiration pneumonia. (Kirk et al., 2009; Honda et al., 2012; Nascimento et al., 2014; Deshwar et al., 2018). This is the most common cause of early death in MDS patients. Some patients have immunoglobulin deficiencies and low antibody titers against pneumococci, making them particularly vulnerable to respiratory infections. (Bauer et al., 2015; Bauer et al., 2018). A few cases of idiopathic pulmonary hypertension have also been reported, indicating that MDS patients are at risk of severe pulmonary complications. (Miguet et al., 2018; Giudice-Nairn et al., 2019). Rett syndrome patients do not exhibit any immune deficiency or recurrent respiratory infections.

Rett syndrome patients often have poor growth, with decreased height and weight. The poor growth may be partially due to altered energy balance and undernutrition due to GI dysfunction. Nutritional intervention (e.g., gastrostomy) can improve growth in patients. (Motil et al., 1994; Motil et al., 2009; Motil et al., 2012). While MDS patients also have GI dysfunction, including feeding difficulties, poor growth has not been reported. Miguet, et al. reported that patients in their cohort were on average of normal height and weight. (Miguet et al., 2018). In our experience, MDS patients also have normal growth including weight, height and occipitofrontal circumference.

Gastrointestinal problems have been extensively compared between MDS and Rett. (Pehlivan et al., 2023). Pehlivan et al. found that MDS individuals more commonly and severely experience GERD (shows bimodal distribution in MDS), chewing difficulty and constipation. On the other hand, air swallowing, excessive gas, and tube feeding dependence are more common in individuals with Rett syndrome.

Many MDS patients have dysmorphic features that have not been described in Rett syndrome patients. This includes large ears, midface hypoplasia, a flat nasal bridge, posterior skull asymmetry, small flat feet, and thin tapered fingers. There have also been a few reports of urogenital abnormalities in MDS patients. (Van Esch et al., 2005; Yi et al., 2016; Miguet et al., 2018; Giudice-Nairn et al., 2019).

Finally, clear genotype/phenotype correlations have been described in Rett syndrome but not MDS. It is known that early, truncating mutations in *MECP2* cause more severe phenotypes in Rett syndrome. (Weaving et al., 2003). There is preliminary evidence that large duplications and translocations outside of the X chromosome cause more severe phenotypes in MDS, but the data has been variable. (Peters et al., 2019; Pascual-Alonso et al., 2020). Clear genotype/phenotype correlations may impact developing treatment options.

## Therapies

Treatment for both conditions currently involves managing symptoms. Treatments for epilepsy, constipation, reflux, and feeding problems is individual-based, and there is no specific treatment for any particular symptom. Physical, occupational and speech therapy help modulate motor and speech symptoms. Vaccines and prompt antibiotic treatment for respiratory infections are especially important in MDS patients. (Van Esch, 2008; Sandweiss et al., 2020). Immunoglobulin therapy and prophylactic antibiotic use can reduce the frequency of infections in MDS. (Bauer et al., 2018). There are no specific recommendations for antiseizure medication for both MDS and Rett syndrome. Our personal experience is that some of the MDS individuals receiving clobazam have worsening of seizures and decreased alertness and strength.

Gene-based therapies are a promising avenue for developing treatment options for *MECP2*-related disorders. It is beginning to be used successfully in other genetic diseases, including spinal muscular atrophy (Mercuri et al., 2018; Al-Zaidy and Mendell, 2019) and Duchenne muscular dystrophy. (McDonald et al., 2017). Studies conducted in mice models of Rett Syndrome (Guy et al., 2007; Garg et al., 2013; Sinnamon et al., 2017; Tillotson et al., 2017) and MDS (Sztainberg et al., 2015) showed phenotype recovery after the genetic aberration was corrected. Endogenous repair of MECP2 mRNA harboring mutations that cause Rett syndrome increased MECP2 protein levels and improved MECP2 function. (Sinnamon et al., 2017). Activation of *MECP2* expression or delivery of *MECP2* via AAV9 to mice with a *MECP2* mutation increased survival, improved the motor phenotype, and reversed the respiratory phenotype back to wild type function. (Guy et al., 2007; Garg et al., 2013; Tillotson et al., 2017). In MDS disease model mice, delivery of ASOs, which alter *MECP2* mRNA processing and decrease protein levels, improved motor function, hypoactivity, anxiety, and social behaviour as well as eliminated seizures. (Sztainberg et al., 2015). A recent study by Shao et al. involved the administration of ASO therapy to mice models expressing two human *MECP2* alleles and no endogenous mice alleles. As before, the ASO therapy improved function and corrected behavioural deficits in a dose-dependent manner without toxicity. (Shao et al., 2021). This recent success suggests that ASO therapy will work in human trials.

There are currently no gene-based therapy treatments approved for use in *MECP2-*related disorders. One challenge is that *MECP2* is a dose-dependent gene. While undertreatment is clearly insufficient, over-treatment will lead to conversion to the opposite disease (Figure 1). ASO therapy, which would be utilized for MDS, requires administration every few months, allowing for changes to the dose if necessary. In contrast, gene replacement therapy in Rett syndrome will be a single dose, rendering any over-treatment irreversible. However, recent developments in gene therapy techniques that increase control of transgene expression have led to the initiation of two clinical trials. Sinnet et al. developed a tool called miRARE that has built in negative feedback regulation to prevent *MECP2* overexpression. (Sinnett et al., 2021). Based on promising pre-clinical results in mice and non-human primates, the REVEAL Adult study will assess the safety, tolerability, and efficacy of the therapy in adult females with Rett syndrome. (Taysha Gene Therapies I, 2023). The company Neurogene has used expression attenuation via construct tuning (EXACT) technology to fine tune transgene expression and reduce the risk of *MECP2* toxicity. In January 2023, Neurogene received clearance from the FDA to start a clinical trial in children with RTT. (Neurogene, 2023).

Another challenge is elucidating the long-term safety of gene therapies. Recent data has triggered concern over the long-term safety of gene replacement therapy via injection of AAV9-SMN in SMA. Studies in mice conducted by Alstyne, et al. found that permanent overexpression of SMN caused sensorimotor deficits, synapse loss, widespread transcriptome abnormalities in dorsal root ganglion neurons, and ultimately neurodegeneration. (Van Alstyne et al., 2021). While the consequences of these findings in SMA patients is unknown, it highlights the complexity of gene therapy and the importance of having a detailed understanding of dose-dependent effects (Crawford and Sumner, 2021; Van Alstyne et al., 2021). Reliable clinical, neurophysiologic and clinical biomarkers are therefore necessary so dosages for each patient can be carefully monitored and titrated.

## Conclusion

The development of safe and effective gene-based therapies for *MECP2*-related disorders requires a comprehensive understanding of both syndromes and the clinical differences between them. Early and accurate diagnoses are necessary, especially in the era of gene therapy when treatment should be administered as rapidly as possible. A more complete clinical picture of MDS is still needed. While cohort studies have worked to describe the clinical features, the relatively small cohorts and differences in study design have made it difficult to establish the prevalence of each feature. A growing understanding and awareness of the disorder along with advances in genetic technology will ideally help fill the gap in knowledge. Gene-based therapies offer a hopeful solution to these disorders that have up to now been untreatable. A thorough understanding of the nuances in clinical phenotype will help advance the development of appropriate treatments.
